# Smart Assistive Technology for Cooking for People With Cognitive Impairments Following a Traumatic Brain Injury: User Experience Study

**DOI:** 10.2196/28701

**Published:** 2022-01-26

**Authors:** Mireille Gagnon-Roy, Stéphanie Pinard, Carolina Bottari, Fanny Le Morellec, Catherine Laliberté, Rym Ben Lagha, Amel Yaddaden, Hélène Pigot, Sylvain Giroux, Nathalie Bier

**Affiliations:** 1 School of Rehabilitation Université de Montréal Montreal, QC Canada; 2 Centre for Interdisciplinary Research in Rehabilitation of Greater Montreal Institut universitaire sur la réadaptation en déficience physique de Montréal du CIUSSS du Centre-Sud-de-l'Île-de-Montréal Montreal, QC Canada; 3 Centre intégré universitaire de santé et de services sociaux de l'Estrie Centre de réadaptation de l'Estrie Sherbrooke, QC Canada; 4 DOMUS Laboratory Department of Computer Science, Faculty of Science Université de Sherbrooke Sherbrooke, QC Canada; 5 Centre de recherche de l’Institut universitaire de gériatrie de Montréal Montreal, QC Canada; 6 Centre de recherche sur le vieillissement Research Center on Aging CSSS-IUGS Sherbrooke, QC Canada

**Keywords:** usability testing and evaluation, user experience, qualitative methods, assistive technologies, rehabilitation, patient safety

## Abstract

**Background:**

User experience (UX), including usability, should be formally assessed multiple times throughout the development process to optimize the acceptability and integration of a new technology before implementing it within the home environment of people living with cognitive impairments.

**Objective:**

The aim of this study is to identify UX issues, notably usability issues, and factors to consider for the future implementation of the COOK (Cognitive Orthosis for Cooking) within the home of individuals with traumatic brain injury (TBI) to identify modifications to improve the technology.

**Methods:**

This study comprised two rounds of UX evaluations, including extensive usability testing, which were completed in a laboratory context: 3 sessions with 5 experts and, after improvement of COOK, 2 sessions with 10 participants with TBI. Each session included the use of scenarios and questionnaires on UX and usability.

**Results:**

Both rounds demonstrated good usability outcomes and hedonic qualities. Various usability issues were identified by participants, such as navigation inconsistencies, technical bugs, and the need for more feedback. Factors to consider in the future implementation of COOK were also mentioned by participants with TBI, including environmental (eg, space available and presence of pets) and personal factors (eg, level of comfort with technology, presence of visual deficits, and preferences).

**Conclusions:**

By evaluating UX, including usability, various times throughout the development process and including experts and end users, our research team was able to develop a technology that was perceived as usable, pleasant, and well-designed. This research is an example of how and when people with cognitive impairments (ie, people with TBI) can be involved in evaluating the UX of new technology.

## Introduction

### Background

Individuals who sustain a traumatic brain injury (TBI) will have to live for numerous years with physical impairments, emotional problems, and cognitive deficits (eg, memory, attention, and executive functions) [1]. These deficits, especially cognitive impairments, may limit their independence and safety in completing everyday activities within their home and community, including instrumental activities of daily living such as meal preparation [2-4]. Indeed, meal preparation involves the coordination of complex tasks using high-level cognitive abilities such as planning, working memory, multitasking, and problem solving, which can be affected in people with TBI [5]. As technology evolves, the use of assistive technology for cognition (ATC) is becoming increasingly attractive to support the functioning of people with TBI [6-9]. For example, De Joode et al [10] demonstrated that a PDA could be as effective as a traditional paper-and-pencil method in achieving personalized goals. Wang et al [11] also compared 2 prompting methods (paper vs via an ATC) during a meal preparation task and showed that prompts provided via an ATC were generally more efficient and appreciated by participants. Therefore, ATCs are a promising avenue for developing and implementing home support interventions for people with cognitive impairments following a TBI. However, to our knowledge, other than the Cueing Kitchen [12,13], which is installed in a laboratory setting, no ATC has been specifically developed to support this population both in terms of safety and independence in meal preparation. The current use of technology to support meal preparation includes the use of reminders and step-by-step instructions [8,14].

In recent years, our interdisciplinary research team (including experts in computer sciences, engineering, occupational therapy, physiotherapy, speech-language pathology, neuropsychology, and evaluative and implementation research) closely collaborated with people who sustained a severe TBI (principal end users), their families, and the team of care (specialized educators, occupational therapists, social workers, and managers) to design an ATC named the COOK (Cognitive Orthosis for Cooking) [15]. Using a user-centered design [16], this cooking assistant was initially developed for 3 persons living with a severe TBI in an alternative housing unit with 24-hour supervision to promote their autonomy and resume meaningful activity (ie, meal preparation) [15,17]. Our research team ultimately aimed to expand its potential to a broader population with TBI (eg, those living in their own apartments in the community). The aim of this paper is to present an overview of the usability evaluation completed throughout the process of developing this technology.

### The Cognitive Assistant—COOK

**Figure 1:**
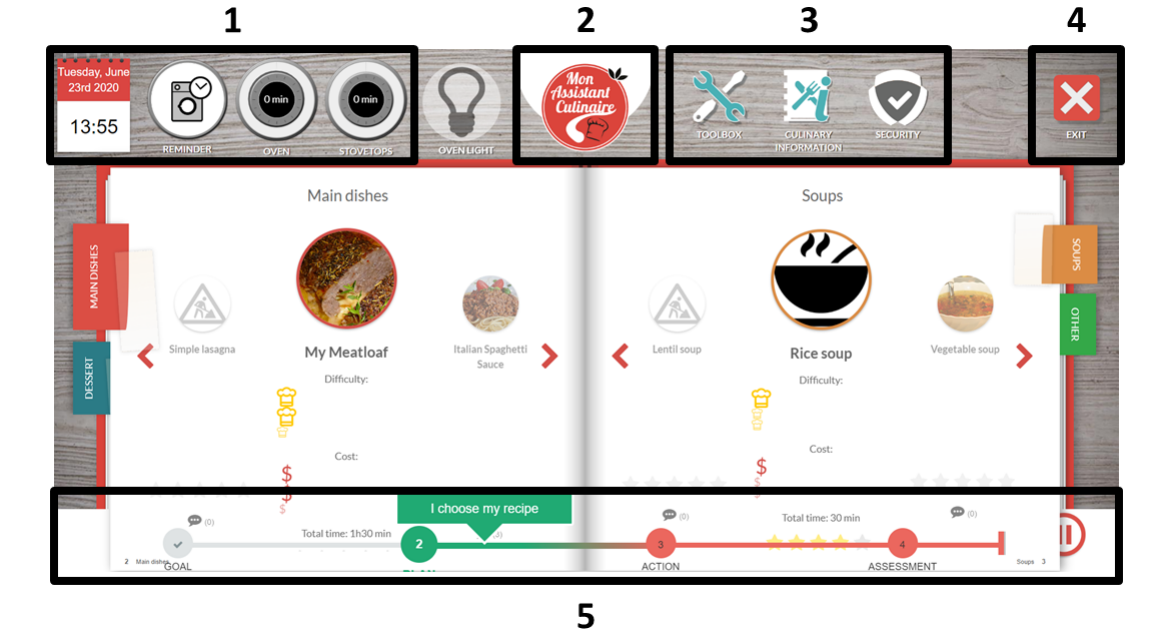
Screenshot of COOK (Cognitive Orthosis for Cooking). (1) Time and date, reminder for another task (eg, washing machine) and timers for the burner and the oven; (2) return to the home page; (3) toolbox (including stress management, notes, and personalized objectives), culinary information (eg, food storage charts, idea of spices, and recommended internal cooking temperatures), and safety rules; (4) exit; (5) steps of the meal preparation task, including goal formulation, planning, conducting the task and self-assessment, and breaks.

**Figure 2:**
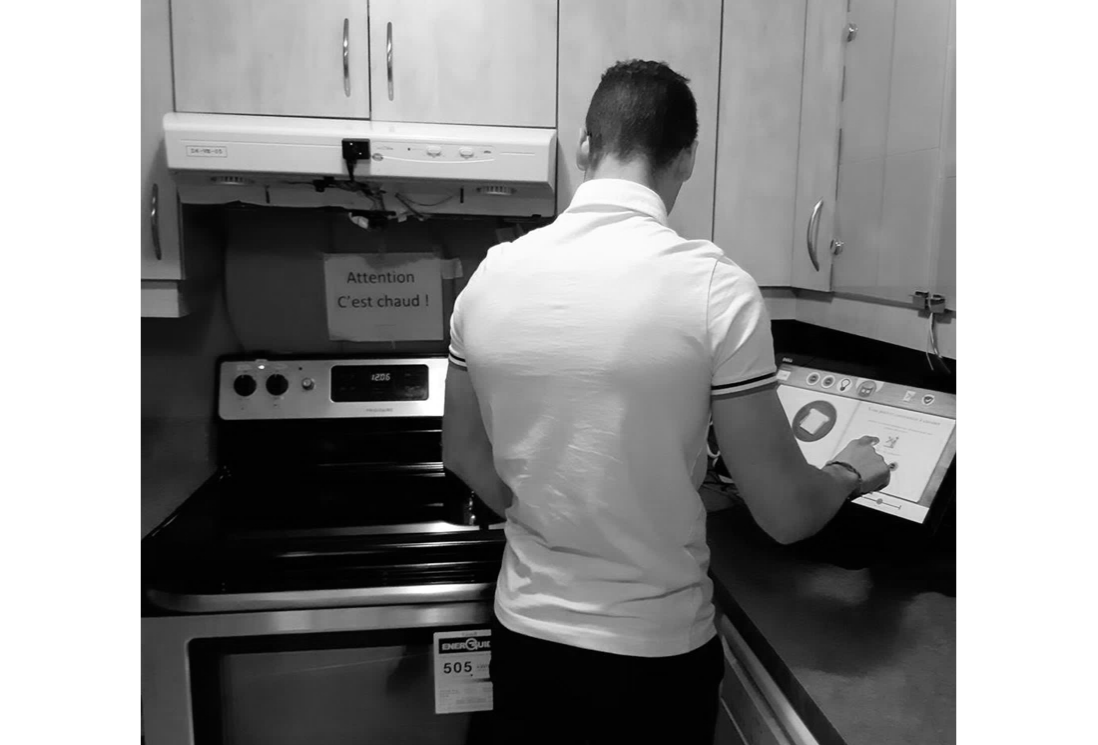
Installation of COOK (Cognitive Orthosis for Cooking) in the laboratory setting.

**Figure 3:**
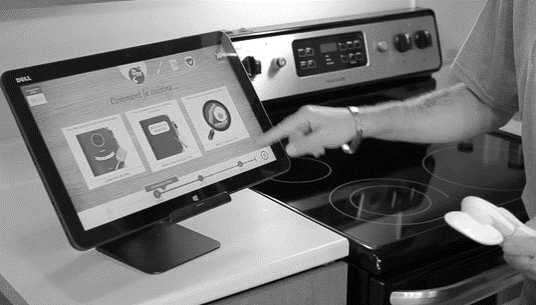
Installation of COOK (Cognitive Orthosis for Cooking) in the laboratory setting.

COOK is a web application that was developed to work on any device with a tactile screen (eg, electronic tablet or computer). For this project, a Dell XPS 18 portable all-in-one desktop computer was used. COOK consists of two systems that work in complementarity: (1) a cognitive support module that guides the user through the interface on the screen (see Figure 1 for an example) and (2) the self-monitoring security system (SSS), which is connected to a smart stove. The cognitive support module encompasses cognitive interventions and functionalities configured by occupational therapists based on their evaluation of the person and the type of intervention approach he or she needs during meal preparation (eg, rehabilitation or compensatory). This can include such things as reminders to reduce distractors and optimize the cooking environment, adapted recipes, food storage charts, timers, and notes. The SSS works with connected sensors installed in the kitchen environment and the smart stove to follow kitchen-related activity and detect at-risk situations (eg, forgetting to turn off a burner). When such situations are detected, the user is warned via the interface and, if he or she does not correct the situation, the stove is automatically shut down. To ensure safety, the use of COOK is required to activate and use the stove. COOK can also be set up according to the user’s needs and preferences. Finally, an interface is available for caregivers to monitor the stove and SSS state (eg, activated or shut down following an at-risk situation). For this study, COOK was installed in a laboratory setting organized as an apartment, including a living room, a bathroom, a main door, and a fully functional kitchen equipped with a smart stove (Figures 2 and 3).

### User Experience Evaluation

**Figure 4:**
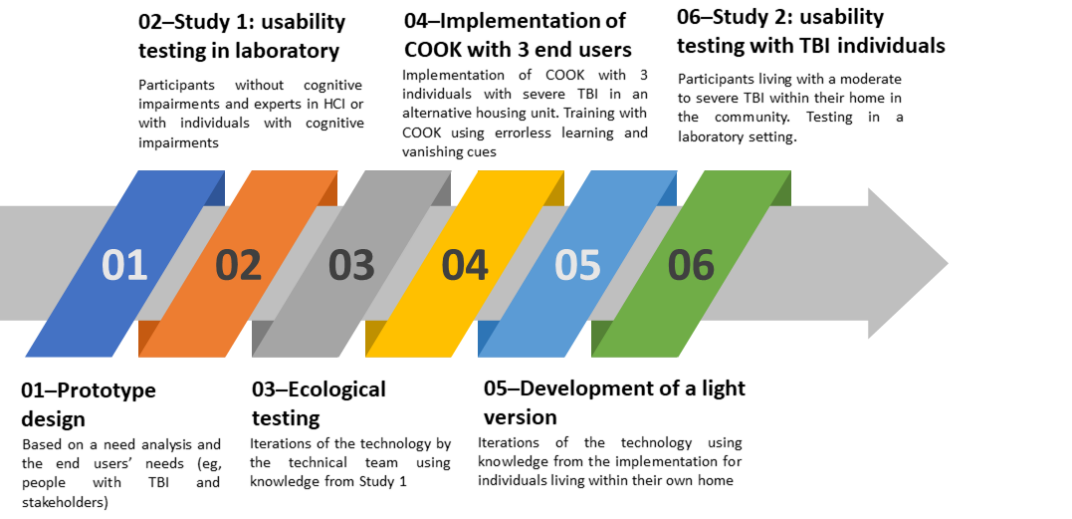
Steps of development and usability tests of the cognitive assistant (COOK). COOK: Cognitive Orthosis for Cooking; HCI: human–computer interaction; TBI: traumatic brain injury.

Previous studies have demonstrated that poor user experience (UX), including poor usability (eg, lack of knowledge and training or improper design according to the user’s needs), was associated with nonadoption of ATCs [18]. Therefore, it was essential to formally evaluate UX at various time points in the development of COOK to develop a more *usable* product [19,20]. UX results from interactions among the user (eg, needs and expectations), the system (eg, functionalities and usability), and the context, and thus considers *hedonic* qualities (eg, pleasure and emotions) [21,22]. Usability, which is an important element contributing to a good UX, refers to the degree to which users are able to attain their goals with efficacy, efficiency, and satisfaction in a specific context using an ATC [23]. As presented in Figure 4, our research team completed 6 broad steps, of which 2 are further explained in this paper (steps 2 and 6). Step 4, which comprises the implementation of COOK with 3 individuals living with a severe TBI in an alternative housing unit and UX evaluation within this real-world environment, is described elsewhere [15].

More specifically, this project aims to (1) document UX issues, particularly usability issues, that could interfere with the use of COOK by individuals living with TBI; (2) identify modifications to improve the technology; and (3) explore factors to consider in the future implementation of COOK within the homes of individuals with TBI.

## Methods

### Study 1: Experts’ Perspective on UX

#### Overview

The first study (step 2 in Figure 4) focused on testing the functionalities of the cooking assistant early in its development process to improve its UX. Considering the end users’ cognitive impairments (eg, limited cognitive load, learning potential, and memory deficits), they are more likely to replicate their mistakes and not be able to correct themselves over time if in contact with a preliminary version of the technology. Therefore, it was preferred to not involve the 3 participants with TBI who participated in step 4 at this step of the development process to reduce risks of integrating faulty ways of using COOK and becoming frustrated as a result. Instead, only individuals with expertise in human-computer interaction (HCI) or with clinical experience with future end users (ie, people living with TBI) were involved in this preliminary step of development as they could provide extensive feedback and potential solutions to the identified UX issues and help our research team reduce bugs and limit future major necessary modifications that could interfere with the further steps in the development process. In the same vein, no caregivers or health providers were included at this step of the project, although they could participate in step 4. This study was approved by the research ethics committee of the Centre Intégré Universitaire en Santé et Services Sociaux of Estrie–Centre hospitalier universitaire de Sherbrooke (CRIR-897-113), and all participants provided their informed consent. A total of three usability tests were conducted in study 1: tests for version 2.1, version 2.2, and a preliminary version of the SSS.

#### Participants

Using convenience sampling, 8 French-speaking individuals with expertise in HCI or clinical experience with clients with cognitive impairments were recruited to participate in at least 1 of the 3 UX tests. Participants were recruited from collaborators involved in other projects conducted at the research laboratory. A clinician specialized in visual impairments was also recruited to obtain her perspective on the visual accessibility of COOK. Among the group of 8 participants, a sample of 5 (63%) participants for each test was considered enough to uncover most UX issues, notably usability issues [24]. Before each UX evaluation, the participants had to complete a 7-point Likert scale, where 1 corresponded to *never* and 7 to *all the tim*e, to measure the extent to which they used electronic tablets on a monthly basis and the number of meals prepared during a week (ie, cooking habits).

#### Task and Procedure

#### Overview

The UX evaluation was completed with 3 tests (1 each) for versions 2.1 and 2.2 and the SSS. Each test included three steps: (1) a general presentation of COOK (including the context of the project and its future use), (2) scenarios simulating the use of the technology during an activity (eg, meal preparation or meal planning, depending on the version tested), and (3) administration of 2 questionnaires measuring usability with the System Usability Scale (SUS) [25-27] and UX from a more global perspective with the AttrakDiff scale [28,29]. All the UX tests were completed between January 2016 and December 2016 and were audiotaped. Each test was completed with the participant, an evaluator, and an observer who took notes.

After the presentation of the cooking assistant, participants were invited to follow scenarios simulating different tasks that could be achieved using COOK. During each simulation, participants were asked to think aloud and describe their thoughts and judgments, explain their understanding of the task and the technology, and comment on the ease of use and potential UX issues and usability issues in particular. As recommended to design technologies, open-ended questions, such as “You seemed surprised, what led you to feel like this?” and “How did you know that you had to...?*”* were also asked to help participants further express their thoughts and actions [30]. All comments from participants were systematically transcribed using observer notes and records, and then deductively regrouped by functionalities and usability issues (eg, size of labels and understanding of messages provided by the technology) to identify the number of times each comment emerged. At this point of development, it was preferred to provide the development team with an exhaustive and detailed list of comments mentioned by the participants to facilitate modifications of the technology. Following UX evaluation, grouped comments were translated into requests, prioritized, and transmitted to the development team to improve the cooking assistant.

Scenarios varied depending on the version assessed in UX evaluation.

#### Version 2.1

Participants were invited to simulate 2 activities of meal preparation (ie, with and without a recipe) and explore functionalities that were developed to help end users follow through the task (eg, timers, culinary information, breaks, and self-assessment).

#### Version 2.2

Participants were invited to simulate a meal preparation activity (ie, with a recipe) to explore new functionalities that had been added in version 2.2 (eg, voice command and vocal synthesis). Participants were also asked to plan meals using COOK.

#### SSS Module

Participants were invited to try the SSS safety rules using 8 scenarios. For 75% (6/8) of the scenarios, participants had to simulate the use of the stove during a meal preparation task while their actions were being supervised by the security system. Each of these scenarios was designed such that a security rule would be triggered. Participants were then asked to react to the various warnings and information transmitted by sound (voice synthesis) and text (pop-up) modalities. In the last 2 scenarios, participants played the role of a caregiver who received notifications about the status of the SSS via a screen in another room. A member of the research team played the role of the person using the stove and needing assistance to restart it after it had been turned off by the SSS.

#### Measures

The following two questionnaires were used: the SUS and the AttrakDiff scale.

The SUS is a highly robust and versatile tool developed by Brooke [25] to evaluate perceived and subjective usability [26]. This questionnaire consists of 10 statements that are scored on a 5-point Likert scale of agreement, with 1 corresponding to *totally disagree* and 5 to *totally agree*. The total score varies between 0 and 100, with higher scores corresponding to stronger usability. The total score can then be qualified using the adjective rating scale (eg, awful, okay, and excellent) to provide a better understanding of the usability value [26,31]. A French translation of the scale was developed by our team and used as no validated version in French was available at the time of the study.

The AttrakDiff is a standardized questionnaire that includes 4 scales with 7 items, totaling to 28 items that evaluate the pragmatic and hedonic qualities of a system [32]. The scales evaluated in the AttrakDiff are the pragmatic quality, hedonic-stimulation quality, hedonic-identity quality, and global attraction. For each scale, an average score varying between –3 and 3 was calculated, where a higher score was associated with positive UX. For this study, the AttrakDiff was an interesting choice to measure UX as it allows comparisons between different versions of a specific product, thus highlighting the potential impact of modifications of COOK on the end users’ experience. The French version of the AttrakDiff was used in this study [28].

### Study 2: People With TBI’s Perspective on UX

#### Overview

In accordance with our goal of expanding the potential use of COOK to a broader population with TBI (including those living within their home in the community), the second round of UX evaluation was completed 3 years after the first study in a laboratory context with participants living with moderate to severe TBI. Despite their cognitive impairments, this step was possible as COOK was previously demonstrated as helpful for 3 individuals with TBI (step 4) by allowing them to prepare 3 meals per week independently and safely [15], and the prototype had since been improved (steps 3 and 5). Moreover, contrary to UX evaluations completed within a real-world context (which involves implementation and training with COOK), UX evaluations in a laboratory could be completed with a larger sample, thus allowing more variability in terms of needs. This study was approved by the ethical review board of the Centre for Interdisciplinary Research in Rehabilitation of Greater Montreal (CRIR-1173-0616). All participants provided their informed consent.

#### Participants

A total of 10 adults living with moderate to severe TBI and interested in meal preparation were recruited to participate in this study. Recruitment was completed in collaboration with rehabilitation centers in and around Montreal and a regional TBI association. Before the first session, each participant was asked questions about his or her TBI (ie, TBI severity and time post injury). They also had to complete the same Likert scale as the one used in the first study to measure their habits (ie, use of an electronic tablet and number of meals prepared during a week) and describe their difficulties.

#### Task and Procedure

#### Overview

UX evaluation was completed in a laboratory setting over two sessions: the first session focused on the SSS, and the second focused on the cognitive support module (including functionalities from versions 2.1 and 2.2). Similar to the first study, each session included three steps: (1) a general presentation of COOK by the evaluator, (2) various guided scenarios simulating the use of the technology during an activity of meal preparation or meal planning, and (3) administration of a French version of the SUS and the AttrakDiff scale. This method was inspired by the *cognitive walkthrough with users* approach, which involves documenting UX and usability outcomes through task performance in specific scenarios using *think-aloud* strategies to document the thoughts and opinions of end users [33]. A complementary semistructured interview of approximately 10 minutes was also conducted at the end of each session to explore their opinions on COOK and facilitators and barriers they perceived regarding the potential use of the technology within their home environment (see Textbox 1 for the questions). The UX evaluation was completed between January 2019 and July 2019 with an evaluator and a research assistant who videotaped the sessions.

**Interview guide for study 2 with participants living with moderate to severe traumatic brain injury.**

**First session**

- How did you find your experience with the COOK (Cognitive Orthosis for Cooking)?
  Elements that you liked
  Elements that you disliked
  Ease of use
  Ease of learning
- How do you think COOK could be improved?

**Second session**

- How do you think COOK could help you with meal preparation?
  How often would you use COOK?
  Confidence in your abilities to use COOK?
- How do you think COOK could interfere with your meal preparation?
- In your opinion, what would be the elements that could make it more difficult to use COOK in your home?
- In your opinion, what would be the elements that could facilitate the use of COOK in your home?

Following an exploration of the cooking assistant, participants were invited to trial various scenarios simulating the use of COOK during a meal preparation task and think aloud about the process (eg, ease of use, potential usability issues, and how they could use the technology within their own living context). Owing to cognitive impairments associated with moderate to severe TBI, all participants were guided by an evaluator (ie, occupational therapist) to ensure progression and help them stay motivated and engaged in the testing when confronted with difficulties with the technology. However, as participants were not expected to learn to use COOK following the UX evaluation, flexibility was provided to allow participants to make mistakes and to allow them to try to correct them by themselves. The scenarios used in this study were similar to those described in the *Task and Procedure* section of Study 1.

#### First Session (SSS)

A total of 7 scenarios were completed to test the safety rules when using the stove, including going out of the apartment.

#### Second Session (Cognitive Support Module)

A total of 3 scenarios were completed to simulate 2 activities of meal preparation (ie, with and without a recipe) and meal planning and explore all the functionalities included in the cooking assistant to help the person complete these tasks.

All sessions were videotaped and transcribed to document observable behaviors (gestures, facial expressions, and automatic reactions) and participant comments. Then, qualitative data (ie, comments and interviews) were analyzed in 2 steps. First, as in study 1, comments specific to COOK’s functionalities were regrouped and translated into requests for the ATC development team to improve COOK. Then, an inductive thematic analysis as described by Miles et al [34] was completed and validated by 2 authors (MGR and RBL) to highlight potential factors that could influence the implementation of COOK within the home of individuals with TBI.

## Results

### Study 1: Experts’ Perspective on UX

#### Overview

Of the 8 participants, 2 (25%) women and 6 (75%) men with expertise in HCI or clinical experience with clients with cognitive impairments, including an expert with 10 years of experience with clients with visual impairments, participated in the UX evaluation. Participants’ characteristics and the UX tests in which they were involved are presented in Table 1. Each UX test lasted between 64 and 113 minutes, with an average of 80.9 minutes per session.

**Table 1:** Participant characteristics and involvement in user experience (UX) tests.

Characteristics			Age (years)		Level of expertise (years)				Electronic tablet use (score)^a^		Cooking habits (score)^a^		UX tests				
					HCI^b^		Cognitive impairments^c^						2.1		2.2		SSS^d^
**Gender**																	
	Male	35		10		0.25		7		3		✓^e^		✓		✓	
	Female	36		0		3		5		7		✓					
	Male	27		3		1		7		4		✓		✓		✓	
	Male	28		2		2		7		3		✓				✓	
	Male	27		17		1		7		5		✓				✓	
	Male	25		3		1		7		3				✓		✓	
	Male	25		8		0		1		5				✓			
	Female	25		3		0.25		1		4				✓			
Values, mean (SD)			28.5 (4.5)		5.75 (5.6)		1.3 (1)		5.25 (2.7)		4.25 (1.4)		N/A^f^		N/A		N/A

^a^A higher score is associated with more frequent use of an electronic tablet and number of meals prepared per week at their entry into the study.

^b^HCI: human-computer interaction.

^c^Cognitive impairments: With a clientele with cognitive impairments.

^d^SSS: self-monitoring security system.

^e^✓: Indicates which UX tests were completed by participants.

^f^N/A: not applicable.

In total, 320 comments were documented and regrouped over the 3 UX tests of the first round, with 155 (48.4%) comments for version 2.1, 53 (16.7%) comments for version 2.2, and 112 (35%) comments for SSS. In response, 108 requests (n=53, 49.1%, n=34, 31.5%, and n=21, 19.4% issues) were translated and transmitted to the development team, of which many were considered and integrated into the next prototype of the cooking assistant. The documented comments encompassed UX and, in particular, usability issues such as navigation inconsistencies (eg, size and location of logos, optimizing navigation between the cooking assistant functionalities, and having access to a search mode to browse through the recipe book), technical bugs, and difficulties of use (eg, with the on-screen keyboard, when writing notes for later use, and with voice command). The need for more feedback (eg, when sending an email) and information (eg, in the recipes, following shut down by the SSS for both the user and the caregiver) was also identified.

#### Questionnaires

Overall, the usability of the preliminary version of COOK was adequate, with scores on the SUS ranging from 79.5 (ie, good usability) to 82.5 (ie, excellent usability) out of 100. The scores are presented in Table 2.

**Table 2:** Scores on the System Usability Scale (SUS) and AttrakDiff for each user experience test.

Questionnaire			Version 2.1, mean (SD)		Version 2.2, mean (SD)		SSS^a^, mean (SD)
SUS^b^ (out of 100)			82 (9.91)		82.5 (2.50)		79.5 (9.10)
**AttrakDiff (between -3 and 3)**							
	PQ^c^	1 (0.40)		1.46 (0.44)		1.49 (0.50)	
	HQ-S^d^	1.63 (0.42)		1.2 (0.67)		1.31 (0.63)	
	HQ-I^e^	1.34 (0.90)		1.4 (0.60)		1.51 (0.55)	
	ATT^f^	2.09 (0.55)		2.03 (0.56)		1.97 (0.62)	

^a^SSS: self-monitoring security system.

^b^SUS: System Usability Scale.

^c^PQ: pragmatic quality.

^d^HQ-S: hedonic-stimulation quality.

^e^HQ-I: hedonic-identity quality.

^f^ATT: global attraction.

**Figure 5:**
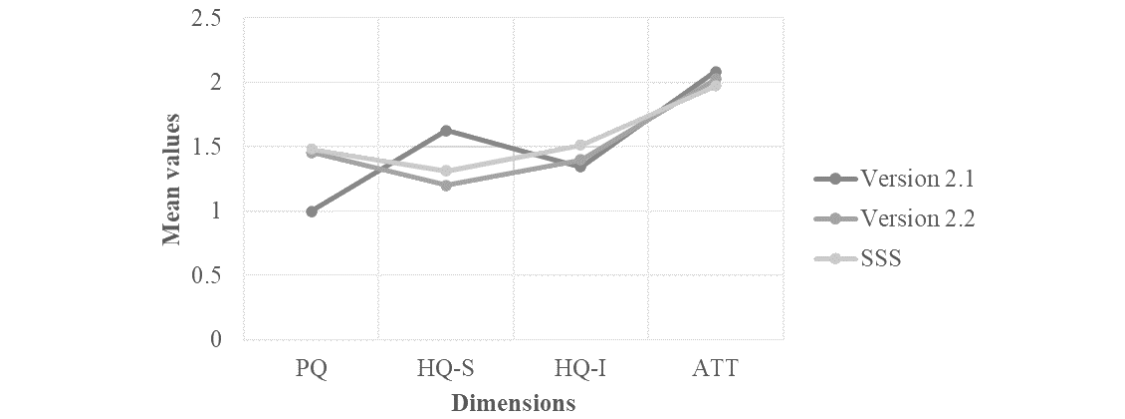
Mean values of the 4 scales of the AttrakDiff for each version that was tested. ATT: global attraction; HQ-I: hedonic-identity quality; HQ-S: hedonic-stimulation quality; PQ: pragmatic quality; SSS: self-monitoring security system.

**Figure 6:**
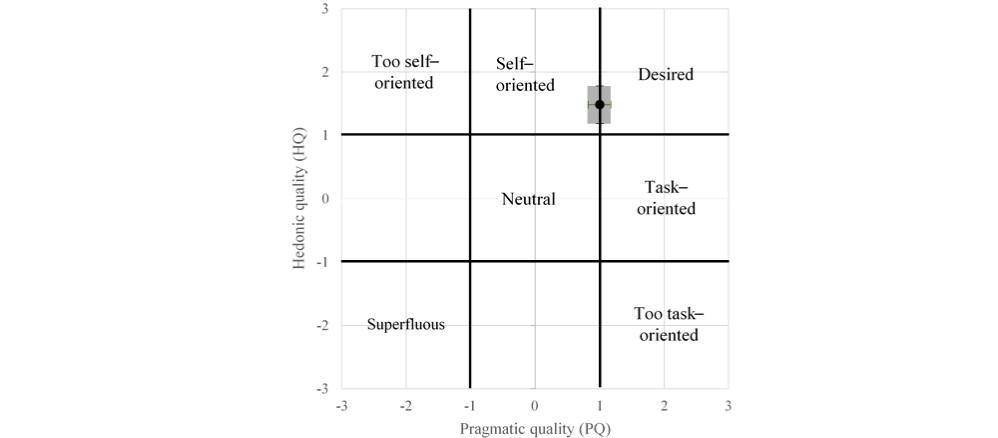
Portfolio of the AttrakDiff–version 2.1.

In terms of UX, all dimensions were identified as positive, as shown in Figure 5. Global attraction was the most positive dimension for all versions of the cooking assistant, whereas the pragmatic quality for version 2.1 received the lowest score. When focusing on the portfolio of the AttrakDiff (see Figure 6 for an example and Multimedia Appendix 1), COOK was overall placed as desired, although version 2.1 also emerged as self-oriented.

### Study 2: People With TBI’s Perspective on UX

#### Overview

A total of 10 participants—3 (30%) women and 7 (70%) men—living with a moderate to severe TBI participated in this study. At the time of the study, all participants had completed or were completing their outpatient rehabilitation. Participants were living in the community within their homes (with or without a family member), except for a participant who was living in a residence. Their age varied between 23 and 61 years (mean 39, SD 11.4 years), and their mean level of education was 12.7 (SD 2.7) years. Of the 10 participants, 2 (20%) had sustained a moderate TBI, and 8 (80%) had a severe TBI, mainly caused by motor vehicle accidents. The mean time post injury was 11.0 (SD 11.8) years (range 1.7-38 years). None of them had returned to work at the time of the study. When questioned about their difficulties when preparing meals and using technologies, the main identified difficulties included visual deficits (eg, sensitivity to blue light), physical impairments (eg, tremors and coordination deficits), cognitive difficulties (eg, fatigue, difficulty with multitasking, and forgetting things), and lack of knowledge and ideas about meals. The participants’ characteristics are presented in Table 3.

**Table 3:** Characteristics of participants living with traumatic brain injury (TBI).

Identifiers and values		Gender	Age (years)	TBI severity		Time post injury (years)	Electronic tablet use (score)^a^	Cooking habits (score)^a^
				Moderate	Severe			
**Participant identifier**								
	1	Male	34		✓	10.7	7	4
	2	Male	23		✓	2.3	6	1
	3	Male	52	✓^b^		38	7	4
	4	Female	30		✓	12.1	5	2
	5	Male	39		✓	2.1	1	2
	6	Female	48		✓	24	1	2
	7	Female	35		✓	2.5	6	2
	8	Male	34		✓	5	1	5
	9	Male	34		✓	1.7	6	6
	10	Male	61	✓		11.2	7	7
Values, mean (SD)		N/A^c^	39 (11.4)	N/A	N/A	11 (11.8)	4.7 (2.6)	3.5 (2)

^a^Higher score is associated with more frequent use of an electronic tablet and number of meals prepared per week (maximum score is 7).

^b^✓: Indicates the TBI severity for each participant.

^c^N/A: not applicable.

226 different comments and observable behaviors were documented over the 2 sessions by participants with TBI (n=48, 21.2% comments for the SSS and n=178, 78.8% comments for the cognitive support module). Many of these comments highlighted potential improvements to COOK (eg, indicating that a burner is empty, listing the tools required for a recipe, and optimizing the functionality to add a recipe), including further improvements to the modifications previously identified in the first study (eg, feedback when sending an email and optimizing the on-screen keyboard). Technical problems also emerged during the UX evaluation, mainly with the SSS (eg, automatic return to the home page and inability to turn on the stove). Moreover, although some participants were able to instinctively use the functionalities of COOK, most participants required assistance and guidance to explore the functionalities during the scenarios (following the general presentation of the technology). Assistance was provided according to the person’s level of ease with the technology, ranging from questions (eg, “What could you use to explore the recipe book?”) and cues (eg, *Explore the left part of the screen*) to physical guidance (eg, pointing to the functionalities). In fact, of the 10 participants, all participants required assistance at least once during the 2 sessions, and 4 (40%) of them were provided continuous assistance throughout the exploration of COOK. Each UX test lasted between 56 and 130 minutes (total duration ranged from 85 to 240 minutes for the 2 sessions), with an average duration of 84 minutes per session (or 151.2 minutes per participant, as 2/10, 20% of them explored all the functionalities in 1 session). The duration varied widely among participants depending on their need for assistance and guidance.

#### Questionnaires

**Figure 7:**
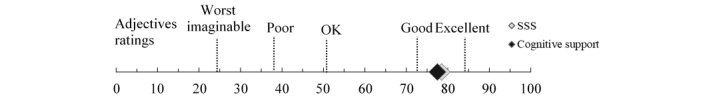
System Usability Scale diagram for the self-monitoring security system and the cognitive support module. SSS: self-monitoring security system.

Regarding usability, the SUS score for the SSS was 78.5 (range 62.5-95) out of 100, and the SUS score for the cognitive support system was 77.5 out of 100. Both scores rated COOK’s usability between good and excellent (Figure 7).

**Figure 8:**
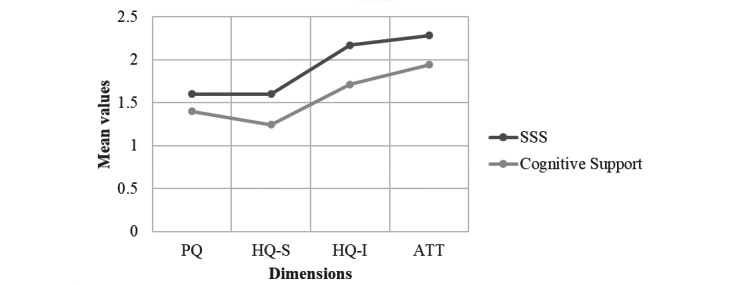
Mean values of the 4 scales of the AttrakDiff for the self-monitoring security system and the cognitive support module. ATT: global attraction; HQ-I: hedonic-identity quality; HQ-S: hedonic-stimulation quality; PQ: pragmatic quality; SSS: self-monitoring security system.

All the dimensions of UX were identified as positive, as shown in Figure 8. Global attraction and the hedonic quality of identity were the most positive dimensions for both systems. Moreover, the SSS system surpassed the cognitive support module for all dimensions of UX, which was coherent with the qualitative feedback that the participants provided during the evaluation sessions. The AttrakDiff also rated COOK as desired in terms of UX (Multimedia Appendix 2).

#### Interviews

Overall, the participants with TBI appreciated both the SSS and the cognitive support system of COOK, describing them as well-made, accessible, and easy to use. In fact, some participants explained that for them, the learning phase could be really short:

I think it’s really obvious. So I don’t think [learning to use COOK] would be problematic, long or arduous.

Participant 9

COOK was also described by participants as helpful for them and others (eg, people with memory deficits and older adults), including for reducing potentially at-risk situations (eg, forgetting the oven or leaving a burner on), helping them return to the task when distracted, and managing meals over the week. For example, a participant explained that she was not cooking without the presence of her spouse because of previously experienced unsafe situations (eg, forgetting something on the stove and burning her meal). Thus, using COOK could allow her to resume meal preparation independently while reducing her spouse’s burden. On the other hand, 20% (2/10) of participants expressed that they would use COOK to help them manage their schedule and find new meal ideas (as they tended to do the same meals over and over again). As a result, 90% (9/10) of the participants mentioned that they would like to have COOK support them at home with meal preparation.

Nonetheless, when discussing the potential use of COOK within their homes, the participants also identified obstacles. First, the participants highlighted that their cooking environment might not be adapted to use COOK. For example, some participants mentioned that they lacked the space to install and use a screen close to the stove:

I am too restricted where I am living, it’s too narrow. [COOK] would be too cumbersome.

Participant 3

The presence of pets was also identified as potentially problematic, as some participants perceived that the sensors could detect their pets in the cooking environment (thus biasing the detection of unsafe behaviors), and the pets could damage electronic equipment (eg, gnaw on the wires). Finally, a participant explained that because of her physical deficits (tremors and having to move around in a wheelchair), her cooking environment was not adapted for her to cook independently using COOK (eg, stove placed too high and lack of support when mixing or stirring her meal). On the other hand, factors related to the participants’ abilities and deficits were also highlighted. Participants mentioned that having difficulties in using everyday technologies (eg, smartphones and computers) could interfere with using COOK and make the learning phase more difficult. For example, a participant explained this as follows:

it's going to take a long time for me to understand the system, how it works, because it's technology, it's something I have trouble with in general.

Participant 4

Visual deficits (eg, difficulty recognizing tools and items in the kitchen, reduced visual acuity, and difficulty finding items in the left space of the screen) were also identified as problematic.

Finally, needs in terms of support for learning were discussed. Many participants highlighted the need for practice, accompanied or not, before being able to use COOK independently within their home environment. Technical support in person or via phone was also mentioned as a requirement following the learning process. Nonetheless, most participants perceived that they could use COOK by themselves with little or no support.

## Discussion

### Principal Findings

The purpose of this paper was to present the results of a UX evaluation completed at various moments throughout the development process of an ATC named COOK. Using similar methodologies, both studies showed that COOK had positive usability outcomes, with SUS scores ranging from good to excellent usability and great UX as assessed using the AttrakDiff scale. Furthermore, both rounds of the study highlighted the potential modifications to COOK. The exploration of COOK in a laboratory setting with participants living with moderate to severe TBI and having various needs and living contexts (eg, living at home with or without a family member or living in a residence) also allowed the identification of factors to consider before using COOK in the community, including space availability in the kitchen, presence of pets, presence of visual deficits, and the person’s level of comfort with everyday technology. Interestingly, although the intention to develop COOK was initially pragmatic (ie, allowing people with TBI to complete a meal preparation task independently and safely and potentially optimizing long-term independence in this task), hedonic qualities emerged as strong in both the studies, which is a positive aspect for future use and implementation of the technology. In fact, awareness of deficits is frequently reduced following a TBI [35,36], and as a result, these individuals often do not perceive the need for cognitive assistance. Consequently, developing a technology that is pleasant, usable, and well-designed, which could ultimately promote acceptability with end users (ie, people with TBI), strongly supported the qualities of COOK for its eventual use.

For this project, the UX evaluation was based on a triangulation of data collection, including standardized questionnaires and the use of scenarios with a *think-aloud* strategy. Although standardized questionnaires allowed comparisons between the versions of COOK and potential users [26,28], using scenarios combined with an explanatory interview emerged as of paramount importance in the process of designing the cooking ATC. First, contrary to the AttrakDiff and SUS, the use of scenarios and analyses of participants’ observable behaviors when following them allowed us to target specific improvements to make to the technology. Second, although most participants with TBI perceived COOK as easy to use and learn (which is coherent with the SUS scores), using a more objective method such as analyzing observable behaviors and assistance provided throughout the scenarios brought to light the extent to which the participants would require a learning phase and support before being able to use COOK independently at home. UX tests were, in fact, conducted by a certified occupational therapist, thus bringing expertise to comprehensively assess a person’s ability to use assistive technology to complete complex activities. Using this expertise, the evaluator was able to provide assistance according to the person’s needs in an informative manner. This is also coherent with prior studies, which suggest that the use of standardized questionnaires or other subjective methods (eg, interviews) as a stand-alone method is not as effective for evaluating UX and its usability outcomes [37,38]. Moreover, very few standardized questionnaires have been developed and validated to evaluate UX of people living with cognitive impairments, such as people with TBI [39]. Thus, the use of both methods was a strength of this project.

### Limitations

Using a triangulation of qualitative methods (eg, scenarios, interviews, and questionnaires), this project demonstrated that COOK has great usability and UX outcomes. Nonetheless, both studies also had some limitations. First, although 5 participants were involved in each UX testing in the first study, only 8 different participants were recruited. As a result, there may have been some learning effect over time, thus influencing the participants’ appreciation of COOK. Nonetheless, new features were tested each time, which likely reduced the learning effect on our results. In addition, although there is a lack of consensus in the literature about the number of participants that should be involved in usability studies, some authors suggest that 5 participants are not enough to identify most usability issues (with identification of only 55% of potential problems in some samples) [40,41]. Nonetheless, we considered that this sample was appropriate for the first study, considering that it was early in the development process and that it included only experts. However, the sample size was larger in the second study as it included participants with cognitive impairments and various needs and living contexts.

By evaluating UX at various times throughout the development process of COOK, our research team was able to obtain a technology that is usable, pleasant, and well-designed while considering the various needs, living contexts, and characteristics of end users (ie, people with TBI). Although other technologies to support meal preparation have been previously developed and tested with people with TBI [11-13], few were formally evaluated in terms of usability and UX. Moreover, in accordance with user-centered design, our research team strongly considered the end users’ needs by including usability evaluation with experts and end users in a laboratory context (study 1 and 2), real-world implementation of the technology [15], and qualitative interviews with stakeholders [42-45], thus contrasting from technologies developed and tested only in a laboratory setting. However, it should be noted that all included participants were adults aged <65 years. Other studies that include older adults are required to explore the UX with COOK in this population as they may experience other obstacles when using technologies [42].

### Conclusions

This paper aimed to present how the UX of different participants when using an ATC for cooking, named COOK, was evaluated in a laboratory context at various times during its development process. Using results from both studies, COOK was improved to facilitate its use by people living with TBI within the community. Factors influencing this process, such as environmental and personal aspects, were identified. Considering the positive appreciation by participants for COOK, further steps should focus on assessing UX when COOK is used within a real-world environment (ie, homes of people with TBI living in the community) and improve its accessibility.

## Appendix

Multimedia Appendix 1 Portfolio of the AttrakDiff for study 1–Version 2.2 and self-monitoring security system.

Multimedia Appendix 2 Portfolio of the AttrakDiff for study 2–Cognitive support module and self-monitoring security system.

## Abbreviations

ATC: assistive technology for cognition
COOK: Cognitive Orthosis for Cooking
FRQS: Fonds de la recherche du Québec–Santé
HCI: human-computer interaction
SSS: self-monitoring security system
SUS: System Usability Scale
TBI: traumatic brain injury
UX: user experience
