# Supplementary material for: Smart Assistive Technology for Cooking for People With Cognitive Impairments Following a Traumatic Brain Injury: User Experience Study
Source: JMIR Rehabil Assist Technol. 2022 Jan 26;9(1):e28701. doi: 10.2196/28701 (PMC8829699; doi:10.2196/28701)
Supplement: Multimedia Appendix 2 [file rehab_v9i1e28701_app2.docx]

Figure S1. *Portfolio of the AttrakDiff for the SSS*

Figure S2. *Portfolio of the AttrakDiff for the cognitive support module*
